# The Effect of NF-κB Signalling Pathway on Expression and Regulation of Nacrein in Pearl Oyster, *Pinctada fucata*

**DOI:** 10.1371/journal.pone.0131711

**Published:** 2015-07-09

**Authors:** Juan Sun, Guangrui Xu, Zeshi Wang, Qing Li, Yu Cui, Liping Xie, Rongqing Zhang

**Affiliations:** 1 Institute of Marine Biotechnology, School of Life Science, Tsinghua University, Beijing, China; 2 Protein Science Laboratory of the Ministry of Education, Tsinghua University, Beijing, China; CAS, CHINA

## Abstract

Nacrein is the first identified and widely investigated molluscan matrix protein and is considered to play an important role in the shell formation of the pearl oyster, *Pinctada fucata*. Here, we investigate the effect of the NF-κB signalling pathway on *Nacrein* gene expression in *P*. *fucata* to elucidate the mechanisms involved in shell formation. Inhibition of NF-κB signalling decreased *Nacrein* promoter-dependent luciferase activity. However, co-transfection of the *Nacrein* promoter vector with Pf-IKK or Pf-Rel expression plasmids could enhance luciferase activity, thus proving NF-κB signalling could regulate the transcriptional activity of the *Nacrein* promoter. Gene silencing by RNA interference and subsequent observation of the inner surface of the nacreous layer of oyster shells by SEM, showed that suppression of the gene *Pf-Rel* lead to a partial inhibition of Nacrein expression, not only at the mRNA level but also at the protein level. The inner surface of the shells became abnormal. Electrophoretic mobility shift assays (EMSAs) revealed that Pf-Rel could directly bind to the relative sites of the *Nacrein* promoter. These results confirm that an important component of the NF-κB signalling pathway, Pf-Rel, can directly bind the *Nacrein* promoter in *P*. *fucata*, and regulate its transcription and shell formation.

## Introduction

Pearl is an example of biomineralization product that has a complicated nacre layer structure. Although accounting for less than 5% of the nacre, matrix proteins control the size and shape of calcium carbonate crystals in pearl and shell formation [1].

By subtle interactions with mineral ion precursors of calcification, such as calcium, bicarbonate and other elements, organic matrix proteins secreted from the mantle are critical for the development of shells in molluscs [2]. These proteins not only participate in the construction of the organic nacre framework but also control the nucleation and growth of aragonitic crystals [3], determining the polymorph specificity of calcium carbonate in nacreous layers. Nacrein was the first matrix protein purified from the nacreous layer within the shell of the pearl oyster. Nacrein was considered to play an important role in the biomineralization process of the shell of *Pinctada fucata* owing to its unique composition, including a domain with homology to carbonic anhydrase and an acidic Gly-Xaa-Asn (Xaa = Asp, Asn, or Glu) calcium binding domain [4]. Nacrein acts as a negative regulator in calcification by inhibiting the precipitation of CaCO_3_ *in vitro*[5] and was involved in ACC and nacreous layer formation in the early formation of pearls [6].

The nuclear factor-κB (NF-κB) signalling pathway, consisting of the core IKK complex, inhibitor IκB protein and transcription factor NF-κB. NF-κB transcription factor was found both in vertebrates [7] and invertebrates [8]. It is known as a classic, evolutionarily conserved, mediator of immune responses in vertebrates [9]. IKK is activated through the effects of many extracellular stimuli and catalyses the phosphorylation, ubiquitination and degradation of IκB proteins, resulting in translocation of the released Rel/NF-κB dimer to the nucleus. After entering the nucleus, the NF-κB/Rel transcription factor binds to specific DNA sequences to regulate gene transcription [10,11].

Since initial discovery as a B-cell-specific transcription factor [12], Previous researches have shown that in mammals, the NF-κB family of transcription factors regulates the expression of a wide array of genes involved in various physiological processes [13–16]. The NF-κB signalling pathway was found not only regulates genes involved in the inflammatory and immune responses, but also plays an important role in bone homeostasis, osteoclast differentiation and vertebrate bone formation [17–20]. Therefore, the NF-κB signalling pathway was thought to be the bridge linking the immune response and bone formation in mammals.

Genes from the pearl oyster *P*. *fucata*, with significant sequence homology to important components of the NF-κB signalling pathway were cloned and named *Pf-IKK*, *Pf-Rel* and *poI*κ*B* [21–23]. Using software[24], we predicted two putative binding sites of NF-κB in the *Nacrein* promoter, suggesting that NF-κB signalling may be involved in regulating *Nacrein* gene transcription. Thus, elucidating any potential regulation of *Nacrein* gene expression by NF-κB signalling could facilitate a better understanding of pearl formation and any interaction that may exist between the immune system and biomineralization in molluscs.

In this study, we investigate the effect of NF-κB signalling on *Nacrein* gene expression in *P*. *fucata*, to further understand the mechanism involved in shell and pearl formation. The transfection of *Pf-IKK* or *Pf-Rel* could increase *Nacrein* promoter-dependent luciferase activity, while the NF-κB inhibitor pyrrolidine dithiocarbamate (PDTC) could decrease luciferase activity. Additionally, RNA interference knockdown of *Pf-Rel* decreased *Nacrein* mRNA levels. Additionally, scanning electron microscopy (SEM) analysis showed a large number of scattered crystal particles appeared on the surface of the nacreous layer after knockdown of *Pf-Rel*, followed by the formation of irregular multi-layer stacking. Electrophoretic mobility shift assays (EMSAs) showed that Pf-Rel could bind directly to the *Nacrein* promoter. These results are consistent with NF-κB regulation of *Nacrein* expression and the shell biomineralization processes in *P*. *fucata*.

## Materials and Methods

### Ethics Statement

This study was approved by the Animal Ethics Committee of Tsinghua University, China.

### Molecular cloning of the *Nacrein* promoter from oyster

We obtained live adult *P*. *fucata* from the Guofa Pearl Farm in Beihai, Guangxi Province, China. The mantle was separated from the oyster with a sterile knife and then ground into very fine powder in liquid nitrogen. Genomic DNA was extracted with the Tissue/Cell genome isolation kit (Tiangen, China) according to the manufacturer’s instructions. A pair of degenerate oligonucleotide primers Nacrein-F and Nacrein-R (Table 1) was synthesized. Using the mantle genome DNA as a template, PCR cycles were carried out in the following steps: denaturation at 95°C for 5 min, followed by 35 cycles of 95°C for 0.5 min, 63°C for 1 min, and 72°C for 4 min. A final extension step was conducted at 72°C for 10 min. The PCR product with the expected size of 1381 bp was confirmed by sequence analysis.

**Table 1:** Nucleotide sequences of primers used in this study.

Gene name	Primer name	Nucleotide sequence
*Nacrein*	Nacrein-F	GGGGTACCTGAGTATCGACGAGAAACGCTTA
	Nacrein-R	CCCAAGCTTCAGTCAAATGAAGATACATCACC
*Pf-Rel*	Rel-exp-F	GCGAGCGAGCGGTGACTTA
	Rel-exp-R	GCACAGGCGCACACATACG
*Pf-Rel*	Sense-Rel	CATCCCCGGGGAGAGCAGCAC
	Antisense-Rel	GTGCTGCTCTCCCCGGGATG
*Nacrein*	Nacrein-RT-F	GGCTTTGGCGACGAACCGGA
	Nacrein-RT-R	ACACGGGGGAGTGGTCAGGG
*Pf-Rel*	Rel-RT-F	CTCGAGTGAAAGCTTCAACA
	Rel-RT-R	CGAGCTATACGAGCACGCAGC
*ß-actin*	actin-RT-F	CTCCTCACTGAAGCCCCCCTCA
	actin-RT-R	ATGGCTGGAATAGGGATTCTGG

### Transient transfection and Luciferase Reporter Assay

#### Construction of vector

We inserted the 1.3 kb PCR-amplified fragment into the multiple cloning site of the pGL3-Basic vector (Promega) to construct the *Nacrein* promoter-luciferase reporter, designated pGL3-*Nacrein*. We used this as a reporter for investigating the activity of *Nacrein* promoter from *P*. *fucata*. Both pcDNA4.0A/*Pf-IKK* and pcDNA4.0A/*Pf-Rel* eukaryotic expression plasmids were constructed following the previously described method for the construction of pcDNA4.0A/*Pf-IKK* plasmid. [21].

#### Transient transfection

24 hours prior to transfection, Hela cells were seeded into 60 mm plates (2–6×10^4^ cells/plate). Cells were transfected with different doses of the reporter luciferase plasmid (pGL3-*Nacrein*), or transfected with the same amount of reporter luciferase plasmid and various amounts of the pcDNA4.0A/*Pf-IKK* or pcDNA4.0A/*Pf-Rel* expression plasmid, with renilla used as an internal reference. The total amount of transfected plasmid was kept constant with the empty expression vector, pGL3-Basic. Transient transfection was carried out using Lipofectamine 2000 Transfection Reagent (Invitrogen) according to the manufacturer’s instructions.

#### Luciferase Reporter Assay

We measured all luciferase activity with a TD-20/20 Luminometer (Promega) according to the manufacturer’s instructions. Reactions were completed in triplicate and statistically significant differences identified by One-way analysis of variance (ANOVA).

#### PDTC inhibition experiments

24 hours after transfection with the reporter luciferase plasmid (pGL3-*Nacrein*), Hela cells were treated with 0–200 μM of PDTC (Sigma-Aldrich) for 24 h before the detection of luciferase activity.

### RNA interference (RNAi)

We used an NCBI blast search to compare the mRNA sequences of *Pf-Rel* for homology with other species. *Pf-Rel* has higher similarity with molluscs than with other unrelated species. A 100 bp sequence in the *Rel* homologous region was highly conservative across all species. *Pf-Rel* silencing probes were designed by GENEIOUS software (Biomatters) as Sense-Rel and Antisense-Rel (Table 1).

Probes were synthesized by Invitrogen, and were diluted in RNase free dH_2_O. *P*. *fucata* with a shell length of 5–6 cm were used in all experiments. 15 ng of dsRNA was injected into the adductor muscle of each oyster by syringe and needle while control samples were injected with 150 mM NaCl. Five individuals were used for each treatment.

### Total RNA extraction and Real-time quantitative PCR

Total RNA was extracted from the oyster mantle 3 or 6 days after injection. Extracted RNA was quantified by absorbance at 260 nm. Quantitative real-time PCR analysis was carried out using 2 μg of total RNA and a Quant Reverse Transcriptase Kit (Tiangen), as per the manufacturer’s instructions.

Real-time PCR analysis was carried out using an Mx3000P RT-PCR System (Stratagene). Target genes were normalized to *ß-actin* mRNA expression levels. The nucleotide sequence of each primer used for real-time PCR is shown in Table 1. PCR amplification was carried out as 95°C for 10 s, followed by 35 cycles of 95°C for 5 s and 60°C for 20 s in duplicate. SYBR Green Real-time PCR Master Mix Kit (TaKaRa) was used for the detection.

All of the real time PCR reactions were repeated in triplicate. The gene expression levels were calculated using the 2^–∆∆Ct^ method [25] and normalized relative to *ß-actin* mRNA at the same time point. The data from the experiments were analysed by ANOVA in Origin 7.0 (OriginLab Corporation).

### Scanning electron microscopic observations

Shells taken from RNAi experiment samples were soaked in 5% NaOH for 8 h to remove organic compounds. Samples were then washed in distilled water several times and air-dried. The nacreous layers were sputter-coated with gold and observed under a QUANTA 200 scanning electron microscope (FEI).

### EMSA

#### Expression and Purification of *Pf-Rel*

*Pf-Rel* cDNA was amplified with a pair of specific primers: Rel-exp-F and Rel-exp-R (Table 1). The PCR products, incorporating BamHI and XhoI restriction sites, were purified, digested and inserted into the prokaryotic expression vector pET28b (Novagen). The recombinant plasmids were confirmed by sequencing.

Remonbiant Pf-Rel was expressed in *Escherichia coli* BL21 (DE3) (Stratagene), following by purification using an AKATA protein purification system with a Ni-NTA column (GE). Fractions of recombinant protein were analyzed by 12% SDS-PAGE. Polyclonal antibodies against Pf-Rel were raised in New Zealand rabbits following standard immunization procedures, and were affinity-purified using the protein A+G-agrose (Beyotime, China), according to the manufacturer’s instructions. The titer was determined using a standard enzyme-linked immunosorbent assay.

#### Nuclear protein extraction

Nuclear protein was extracted from the gills of *P*. *fucata* oysters using a total nuclear protein extraction kit (Xinghan) according to the manufacturer’s instructions. Quantification of nuclear protein was carried out using the bicinchoninic acid method [26].

#### EMSA

EMSAs were carried out using DIG Gel Shift Kit, 2nd Generation (Roche), according to the manufacturer’s instructions. Briefly, the probe containing the consensus Pf-Rel binding site (underlined letters) KBWTL-5: GATCACTGCAACAGGGTCTGGTTGGAGATCCCCTCCCTTCTTGTAAGA was synthesized and labelled with DIG-11-ddUTP. 1 mg of nuclear extract was incubated with the DNA probe at 30°C for 30 min and separated on a 5% native polyacrylamide gel. After electrophoresis, the DNA-protein complexes were blotted onto a nylon^+^ membrane by electro-blotting. The signals were visualized by chemiluminescent detection on X-ray film.

## Results

### *Nacrein* promoter is transcriptionally active

**Fig 1:**
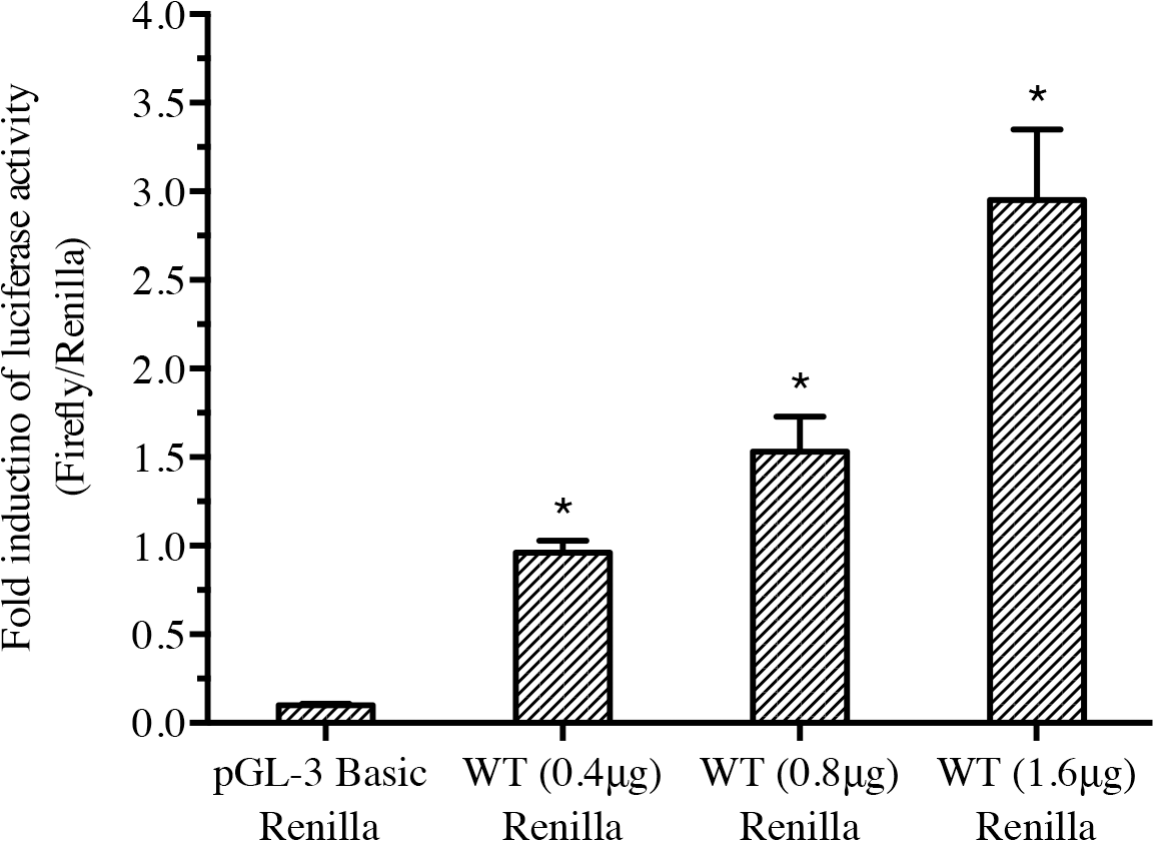
*Nacrein* promoter is transcriptionally active. Different amounts of pGL3-*Nacrein* plasmid (0, 0.4 μg, 0.8 μg, 1.6 μg) was transfected into Hela cells and promoter-dependent Luciferase activity was measured. We used Renilla as an internal reference. Empty pGL-3 Basic plasmid was used to keep the total amount of plasmid the same in each group. Each reaction was completed in triplicate. Luciferase activity increased as the dose of pGL3-*Nacrein* luciferase vector increased, confirming that the constructed pGL3-*Nacrein* promoter plasmid is transcriptionally active. Significant differences were identified by One-way ANOVA. The symbol “*” indicates a significant reduction (P < 0.05), compared to control, which was transfected with pGL-3 Basic alone.

We confirmed transcription of the constructed *Nacrein* promoter plasmid through luciferase activity. Different amounts of pGL3-*Nacrein* luciferase plasmids were transfected into Hela cells and the activity of the *Nacrein* promoter was measured (Fig 1). Luciferase activity increased as the dose of pGL3-*Nacrein* luciferase vector increased, confirming that the constructed pGL3-*Nacrein* promoter plasmid is transcriptionally active and can be used for further transfection experiments.

### Pf-IKK and Pf-Rel increased the *Nacrein* promoter activity

**Fig 2:**
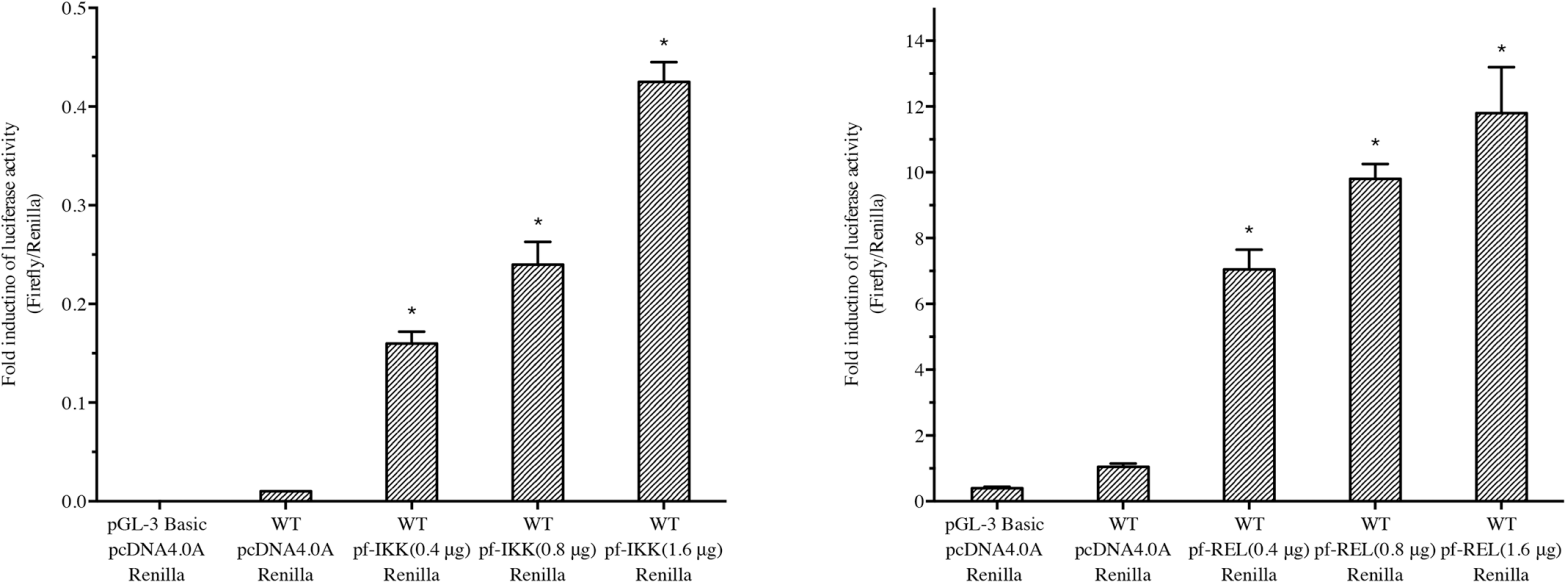
Pf-IKK and Pf-Rel increased the *Nacrein* promoter activity. Increasing doses (0, 0.4 μg, 0.8 μg and 1.6 μg) of pcDNA4.0A/*Pf-IKK* (A) or pcDNA4.0A/*Pf-Rel* (B) were co-transfected with pGL3-*Nacrein* promoter Luciferase plasmids into Hela cells. 0.5 ng of Renilla was used as an internal reference in each group. Empty pcDNA4.0A plasmid was used to keep the total amount of plasmid the same between treatments. Control cells were transfected with pGL3 Basic alone. The luciferase activity, which reports the *Nacrein* promoter activity, increased significantly as the does of (left) *Pf-IKK* or *Pf-Rel* (right) plasmids increased. Significant difference was identified by One-way ANOVA. The symbol “*” indicates a significant reduction (P < 0.05), compared to control.

Both IKK and Rel are important components in the NF-κB pathway, and their homologues, Pf-IKK and Pf-Rel from *P*. *fucata*, have been cloned previously [21,22]. To investigate whether the NF-κB pathway regulates *Nacrein* gene transcription, pGL3-*Nacrein* promoter reporter plasmid was transfect into Hela cells together with pcDNA4.0A/*Pf-IKK* or pcDNA4.0A/*Pf-Rel* plasmids, and Luciferase activity was measured. Increasing concentrations of both the *Pf-IKK* and *Pf-Rel* plasmids increased the activity of the *Nacrein* promoter significantly (Fig 2). This dose dependent increase in luciferase activity shows that the NF-κB signalling is capable of regulating the transcription activity of the *Nacrein* promoter.

### The NF-κB inhibitor PDTC could inhibit the *Nacrein* promoter activity

**Fig 3:**
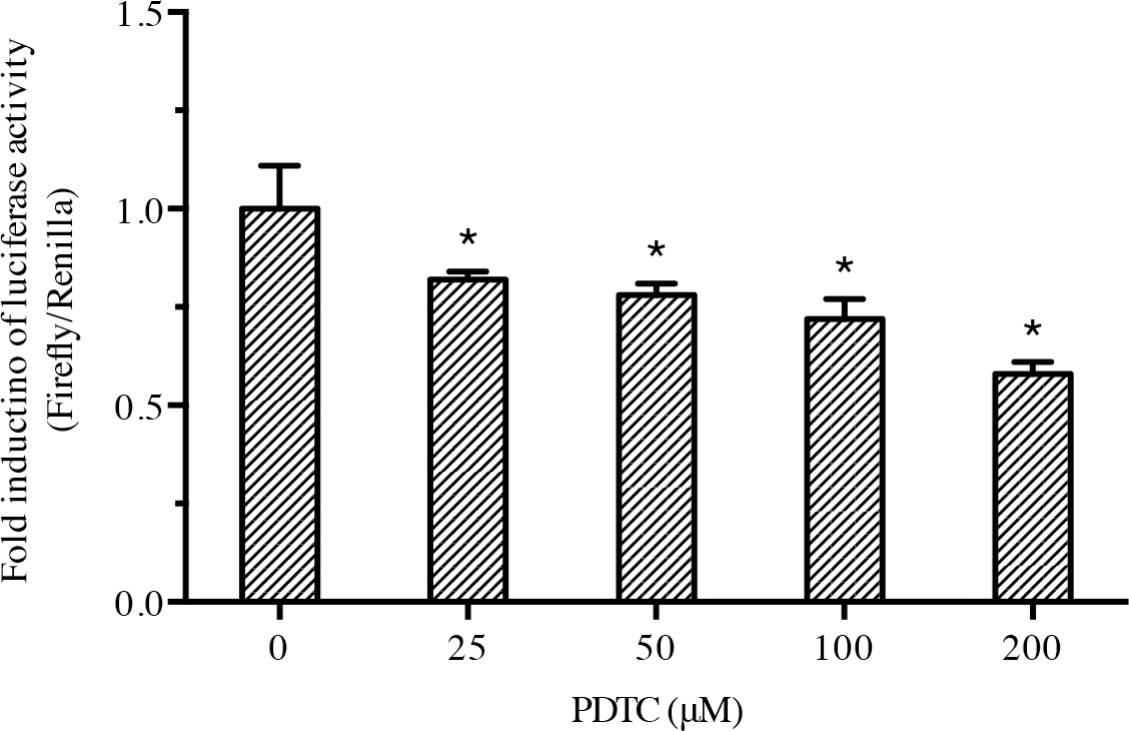
PDTC inhibits the *Nacrein* promoter activity. Equal amounts of pGL3-*Nacrein* Luciferase and pcDNA4.0A/*Pf-IKK* plasmids were co-transfected into Hela cells that were 24 h previously treated with increasing concentrations of PDTC (0, 25 μM, 50 μM, 100 μM and 200 μM). 0.5 ng of Renilla was used as an internal reference. Each reaction was repeated in triplicate. Increasing amounts of PDTC lead to a decrease in luciferase activity, suggesting a decrease in *Nacrein* promoter activity. Significant differences were identified by One-way ANOVA. The symbol “*” indicates a significant reduction (P < 0.05), compared to the control.

PDTC, a metal chelator and antioxidant, can specifically inhibit the activation of NF-κB by suppressing the release of the inhibitory subunit IκB from the latent cytoplasmic form of NF-κB [27]. In order to study the effect of NF-κB signalling on activation of the *Nacrein* promoter, PDTC was added into cultured Hela cells that were co-transfected with the Nacrein-Luciferase reporter plasmid pGL3-*Nacrein* and the plasmid pcDNA4.0A/*Pf-IKK*. The pcDNA4.0A/*Pf-IKK* plasmid was used to enhance the basic activity of the NF-κB promoter. Interestingly, increasing amounts of PDTC lead to a decrease in luciferase activity (Fig 3). This result suggested that the NF-κB signalling pathway was involved in regulating *Nacrein* gene expression.

### *Pf-Rel* knockdown decreased the *Nacrein* gene expression level

**Fig 4:**
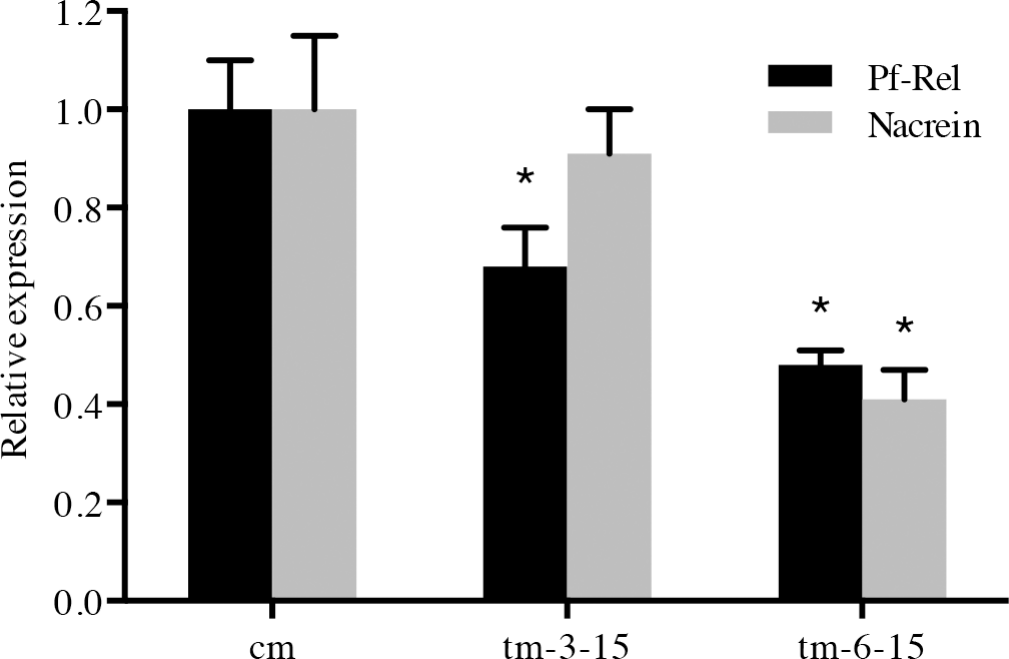
*Pf-Rel* knockdown decreased the *Nacrein* gene expression level. The expression levels of *Nacrein* (grey columns) and *Pf-Rel* mRNA (black columns) in oyster mantle were measured by Real-time PCR, 3 or 6 days after injection of *Pf-Rel* dsRNA. Five oysters (n = 5) were used in each experiment. Reactions were completed in triplicate. cm: NaCl control solution. Both *Nacrein* and *Pf-Rel* mRNA expression level in controls are attributed a relative value of 1.0. tm-3-15 and tm-6-15: samples injected with15 ng *Pif-Rel* dsRNA 3 or 6 days respectively. The expression levels of *Nacrein* slightly decreased after dsRNA injection, compared to the control group. 6 days after injection, the expression level of both *Pf-Rel* and *Nacrein* were suppressed by nearly 50%. Significant difference was identified by One-way ANOVA. The symbol“*”indicates a significant reduction (P < 0.05), compared to control oysters.

The luciferase reporter assay showed that key components in the NF-κB signalling pathway could affect the activity of the *Nacrein* promoter. In order to further clarify if and how NF-κB signalling regulates the *Nacrein* gene transcription, we performed a knockdown of *Rel* gene by RNAi. *Pf-Rel* targeted double strand RNA (dsRNA) was injected into the adductor muscle of *P*. *fucata*. 3 and 6 days after injection, the expression levels of *Pf-Rel* and *Nacrein* mRNA in the oyster mantle were measured by real-time PCR. The expression levels of *Nacrein* slightly decreased after dsRNA injection (Fig 4), compared to the control group (treated with NaCl solution). 6 days after injection, the expression level of both *Pf-Rel* and *Nacrein* were suppressed by nearly 50%. These results demonstrate that silencing of the *Pf-Rel* gene is capable of decreasing *Nacrein* mRNA transcription levels.

### *Pf-Rel* knockdown disturbed the shell biomineralization

**Fig 5:**
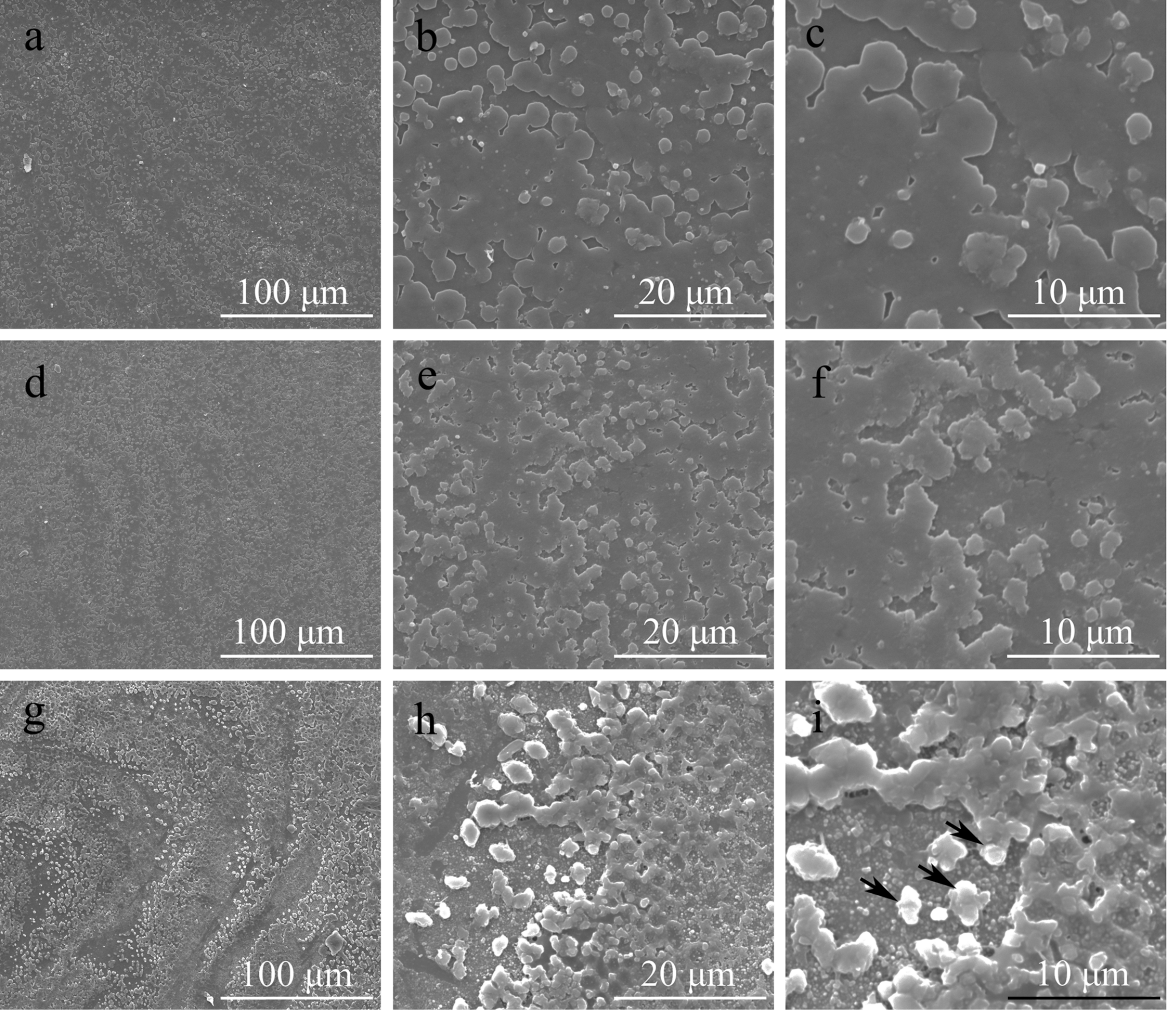
*Pf-Rel* knockdown disturbed the shell biomineralization (SEM). The oysters used were treated for Pf-Rel RNAi by being injected with either NaCl solution or 15 ng Pf-Rel dsRNA. a, b, c: control group with NaCl solution. d, e, f: 3 days after injection with 15 ng dsRNA. g, h, i: 6 days after injection with 15 ng dsRNA. b, e, h: magnified images of a, d, g. c, f, i: magnified images of b, e, h. The black arrows in I indicate the examples of the irregular, multi-layer stacking particles. After 3 days injection (d, e, f), crystal particles became more intensive then the controls. The distribution and the edges of these particles become irregular. After 6 days injection, a large number of scattered crystal particles appeared on the surface of the nacreous shell, followed by the formation of irregular, multi-layer stacking, leading to the complete interruption of the normal layered structure. Scal bars: a, d, g: 100 μm; b, e, h: 20 μm; c, f, i: 10 μm.

As Nacrein plays an important role in pearl biomineralization, a decrease in Nacrein expression could possibly cause changes in the crystal morphology of oyster shells. Therefore, we observed the surface structure of the nacreous layer in each dsRNA injection group using SEM. Compared to NaCl injected controls, oysters with decreased *Nacrein* transcription had an obvious change on the surface of nacreous shell, which was shown 3 and 6 days after injection with 15 μg of *Pf-Rel* dsRNA (Fig 5). After 3 days injection (Fig 5D, 5E and 5F), crystal particles became more intensive then the controls (Fig 5A, 5B and 5C). Their distribution were scattered (Fig 5D and 5E). And the edges of these particles also become irregular (Fig 5F). After 6 days injection, the situation got more serious (Fig 5G, 5H and 5I). A large number of scattered crystal particles appeared on the surface of the nacreous shell, followed by the formation of irregular, multi-layer stacking (Fig 5I), leading to the complete interruption of the normal layered structure. These results are similar to the SEM pictures of *P*. *fucata* nacre shells in which Nacrein was directly inhibited by application of Nacrein monoclonal antibodies [28,29].

### Pf-Rel could bind to the promoter of *Nacrein*

**Fig 6:**
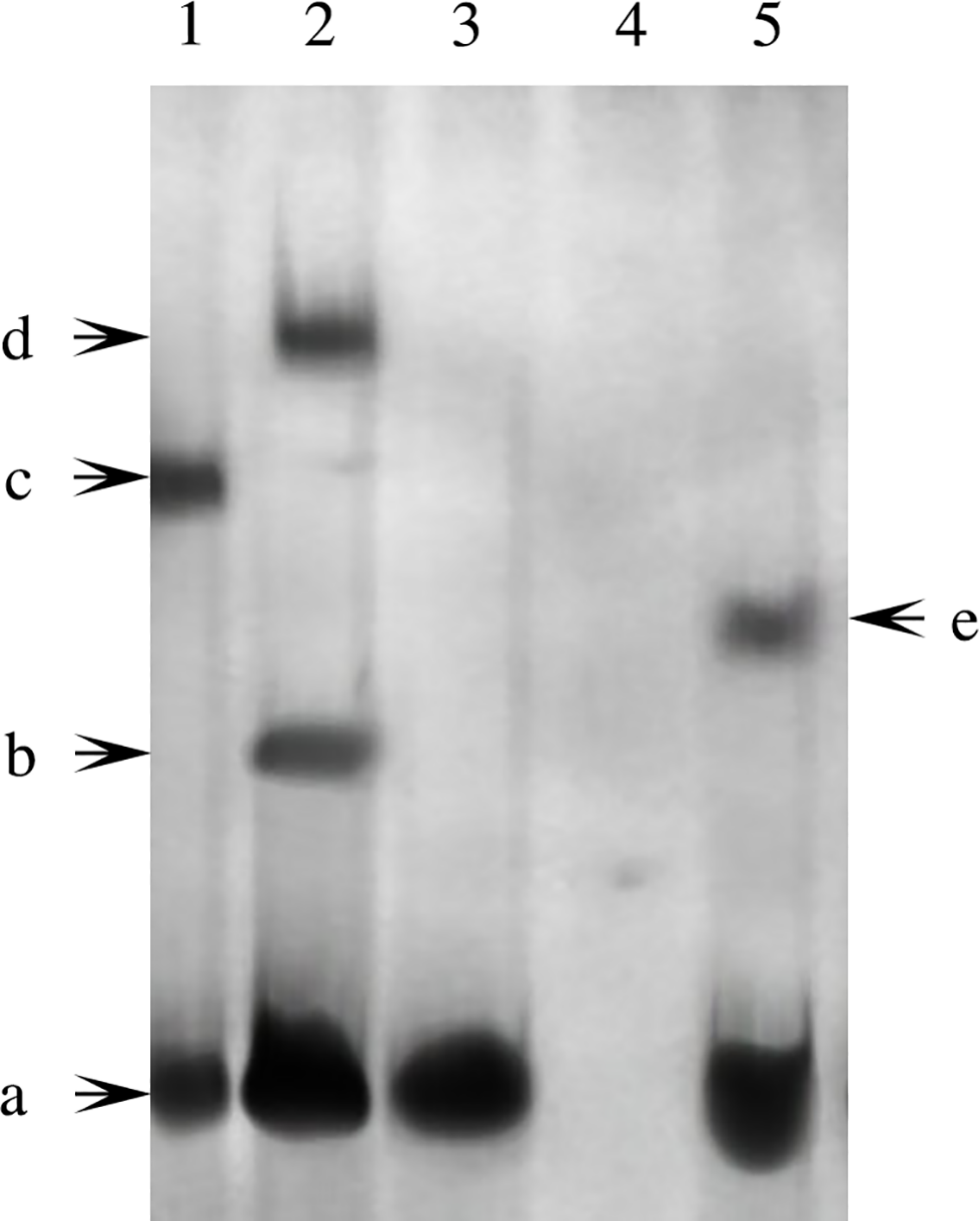
Pf-Rel could bind to the promoter of *Nacrein* (EMSA). Nuclear proteins extracted from the gills of *P*. *fucata* were incubated with oligonucleotide probes labelled with DIG-ddNTP to perform EMSAs. Lane 1: labelled DNA probes, nuclear protein and antibody of Pf-Rel. Lane 2: labelled DNA probes and nuclear protein. Lane 3: labelled DNA probes. Lane 4: unlabelled DNA probes and nuclear protein. Lane 5: positive control. Band a: free DNA. Band b: DNA and Pf-Rel. Band c: DNA, Pf-Rel and antibody of Pf-Rel. Band d: DNA, Pf-Rel and other interactive nuclear protein. Band e: positive control band. Compared to Lane 2, there is a super shift after Pf-Rel antibody was added in Lane 1, suggesting that Pf-Rel present in the total nuclear protein extracts is capable of binding to the *Nacrein* promoter probes.

Through sequence analysis, we identified two possible NF-κB binding sites in the *Nacrein* gene promoter (see S1 Fig). As a putative Rel/NF-κB homolog, Pf-Rel may bind these possible NF-κB binding sites. We performed a series of EMSAs to determine whether Pf-Rel is involved in the nuclear translocation of NF-κB. Nuclear proteins extracted from the gills of *P*. *fucata* were incubated with DIG-labelled *Nacrein* promoter probes and Pf-Rel antibodies. The samples were separated on a non-denaturing PAGE gel and bands were visualized by chemiluminescent detection on X-ray film. As shown in Fig 6, besides Band a (free DNA), no band was detected in Lane 3 and Lane 4. Compared to Lane 2, there is a super shift in Lane 1 after Pf-Rel antibody was added, suggesting that Pf-Rel present in the total nuclear protein extracts is capable of binding to the *Nacrein* promoter probes.

## Discussion

Nacrein is an important matrix protein capable of regulating the formation of oyster shells [4]. In this study we describe how the oyster *P*. *fucata* regulates this important matrix protein both *in vitro* and *in vivo*, and describe the morphological effect of decreased Nacrein expression on oyster shell structure.

Xiong *et al*. found that Pf-IKK activated the expression of NF-κB-controlled reporter genes and induced NF-κB translocation [21]. Sequence analysis of Pf-Rel shows that it shares high similarity with other Rel/NF-κB family proteins, especially within conserved domains [22]. A conserved degradation motif and six ankyrin repeats were identified in the poIκB, which shares significant homology with other IκB proteins [23]. Here, we have shown that the NF-κB signalling pathway exists and functions to regulate *Nacrein* transcription in *P*. *fucata*.

An *in silico* analysis of the *Nacrein* promoter sequence identified two possible NF-κB binding sites between nucleotides 429–438 and 882–891 (see S1 Fig). We were able to confirm that the NF-κB signalling pathway could regulate the activity of the *Nacrein* promoter in *P*. *fucata* by co-transfection PGL3-Nacrein and pcDNA4.0A/*Pf-IKK* or pcDNA4.0A/*Pf-Rel*. Expression of both of these proteins promoted *Nacrein* transcription, while the NF-κB inhibitor PDTC inhibited *Nacrein* transcription.

Although NF-κB signalling is able to regulate the *Nacrein* promoter, we were not sure whether this pathway could regulate the expression level of Nacrein. Real time quantitative PCR results showed that mRNA transcription of *Nacrein* is depressed when *Pf-Rel* mRNA is partially silenced, suggesting that Pf-Rel indeed is involved in regulating *Nacrein* transcription. Significantly, SEM images of the inner nacreous layer structure of shells after dsRNA *Pf-Rel* knockdown showed similar morphological changes to the nacreous layer when Nacrein was inhibited directly by its antibody [28]. EMSAs were able to demonstrate direct binding between Pf-Rel and the *Nacrein* promoter. It therefore appears likely that Pf-Rel regulates *Nacrein* transcription by binding to its promoter.

The NF-κB signalling pathway has been extensively studied in mammals. Here, we focused on the effect of one component of NF-κB signalling in regulating *Nacrein* transcription in pearl oysters. In the future, it will be interesting to investigate if an interaction exists between the different NF-κB signalling components Pf-IKK, poIκB, and Pf-Rel. Because of a lack of mollusc cell lines, transfection experiments have to be performed on mammalian cells instead. It would be interesting to confirm these results experimentally within primary cell cultures of oyster tissue.

## Conclusion

In conclusion, we have shown how the important NF-κB signalling pathway protein Pf-Rel regulates the transcription of *Nacrein*. We described novel NF-κB signalling in *P*. *fucata*, which has the ability to regulate the pearl biomineralization process.

## Supporting Information

S1 Fig Schematic representation of the two NF-κB binding sites in the Nacrein promoter. The black boxes indicates the two NF-κB binding sites in the Nacrein promoter (GeneBank Number AB274024). The binding sites were predicted by the software TF SEARCH[30]. The scores were both 86.8.
(TIF)
