# Supplementary figures and images for: The Effect of NF-κB Signalling Pathway on Expression and Regulation of Nacrein in Pearl Oyster, Pinctada fucata
Source: PLoS One. 2015 Jul 9;10(7):e0131711. doi: 10.1371/journal.pone.0131711 (PMC4497715; doi:10.1371/journal.pone.0131711)

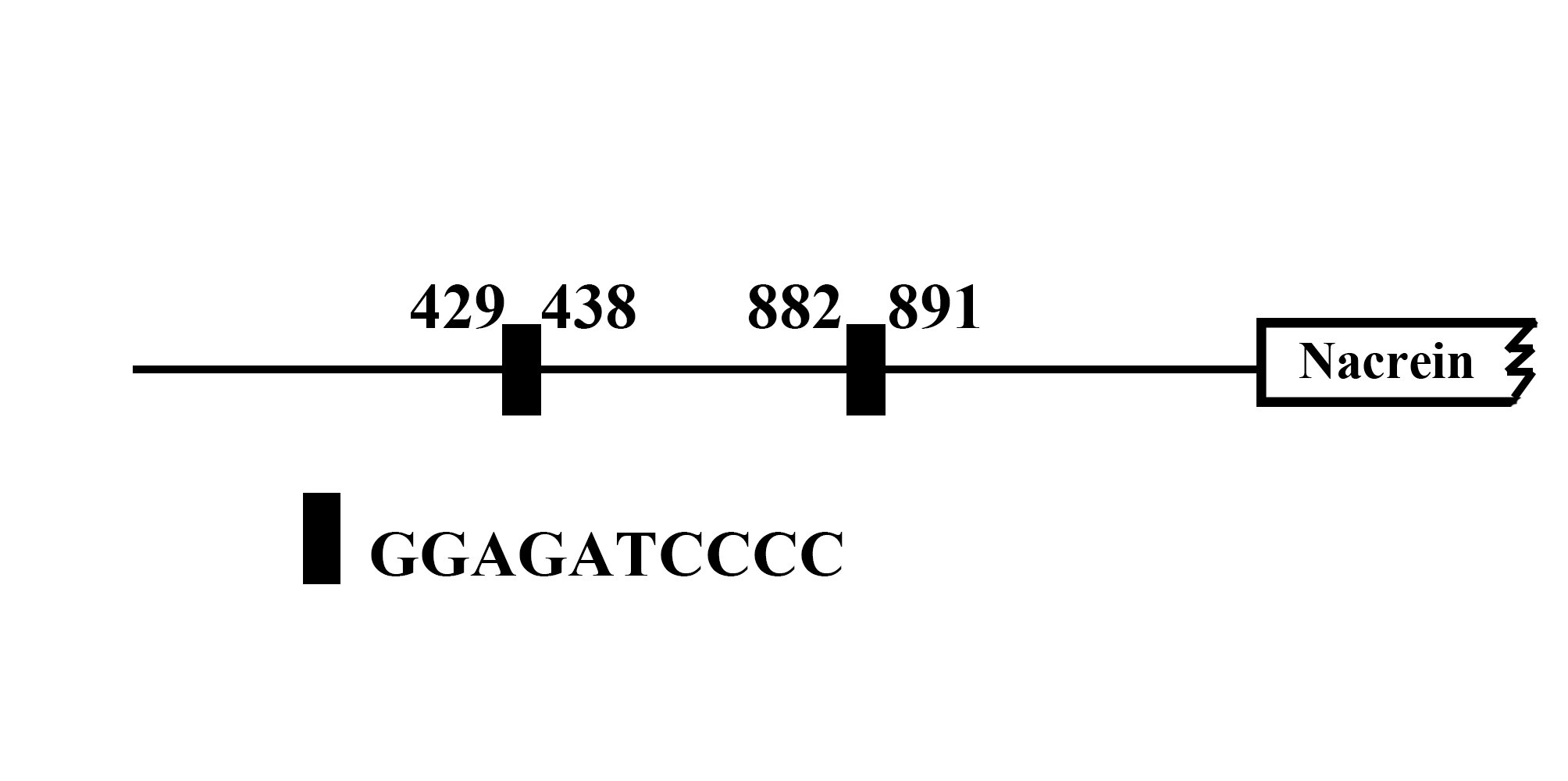

Supplement: S1 Fig — The black boxes indicates the two NF-κB binding sites in the Nacrein promoter (GeneBank Number AB274024). The binding sites were predicted by the software TF SEARCH[30]. The scores were both 86.8. (TIF) [file pone.0131711.s001.tif]
